# ﻿Three new species of *Iodosphaeria* (Xylariomycetidae): *I.chiayiensis*, *I.jinghongensis* and *I.thailandica*

**DOI:** 10.3897/mycokeys.86.75801

**Published:** 2022-01-07

**Authors:** Lakmali S. Dissanayake, Diana S. Marasinghe, Milan C. Samarakoon, Sajeewa S.N. Maharachchikumbura, Peter E. Mortimer, Kevin D. Hyde, Chang-Hsin Kuo, Ji-Chuan Kang

**Affiliations:** 1 Engineering and Research Center for Southwest Bio-Pharmaceutical Resources of National Education Ministry of China, Guizhou University, Guiyang 550025, China Guizhou University Guiyang China; 2 Center of Excellence in Fungal Research, Mae Fah Luang University, Chiang Rai 57100, Thailand Mae Fah Luang University Chiang Rai Thailand; 3 School of Life Science and Technology, University of Electronic Science and Technology of China, Chengdu 611731, China National Chiayi University Chiayi Taiwan; 4 Centre for Mountain Futures (CMF), Kunming Institute of Botany, Honghe County 654400, Yunnan, China University of Electronic Science and Technology of China Chengdu China; 5 Innovative Institute for Plant Health, Zhongkai University of Agriculture and Engineering, Guangzhou 510225, China Kunming Institute of Botany Yunnan China; 6 Department of Plant Medicine, National Chiayi University, 300 Syuefu Road, Chiayi City 60004, Taiwan Zhongkai University of Agriculture and Engineering Guangzhou China

**Keywords:** Ceratosporium-like asexual morph, Sordariomycetes, taxonomy, three new taxa

## Abstract

Three fungal specimens (two sexual and one asexual) were collected during fieldwork conducted in China, Taiwan and Thailand. Both sexual morphs share superficial, black ascomata surrounded by flexuous setae; 8-spored, unitunicate, cylindrical asci, with J+, apical ring, and ellipsoidal to allantoid, aseptate, guttulate ascospores. The asexual morph has ceratosporium-like conidia arising from aerial hyphae with a single arm and are usually attached or with 2–3 arms, brown, often with a subglobose to conical cell at the point of attachment. Morphological examinations and phylogenetic analyses of a combined LSU-ITS dataset *via* maximum likelihood and Bayesian analyses indicated that these three collections were new species. *Iodosphaeriachiayiensis* (sexual morph), *I.thailandica* (sexual morph) and *I.jinghongensis* (asexual morph) are therefore introduced as new species in this study. *Iodosphaeriachiayiensis* has small, hyaline and ellipsoidal to allantoid ascospores, while *I.thailandica* has large ascomata, cylindrical to allantoid asci and hyaline to pale brown ascospores.

## ﻿Introduction

*Iodosphaeria* was introduced by Samuels et al. (1987) with its type *I.phyllophila* on a rachis of *Cyathea* sp., from Brazil. Only five of the nine *Iodosphaeria* species have been sequenced (Li et al. 2015; Marasinghe et al. 2019; Miller and Réblová 2021) and several species of them lack DNA-based sequence data. The sexual morph of *Iodosphaeria* is characterized by superficial, black, apapillate ascomata with unbranched, brown radial flexuous hairs, a two layered peridium composed of a pigmented outer layer and a hyaline inner layer; unitunicate, amyloid or non-amyloid, cylindrical to narrowly clavate, 8-spored asci; and mostly allantoid to ellipsoidal, aseptate, hyaline ascospores with or without a gelatinous sheath (Miller and Réblová 2021). The asexual morphs of *Iodosphaeria* are considered selenosporella-like or ceratosporium-like (Samuels et al. 1987; Li et al. 2015; Miller and Réblová 2021). Members of *Iodosphaeria* are regarded as cosmopolitan species (Li et al. 2015). These species are usually saprobic on dead branches, twigs, stems, and petioles of economically important plants, such as *Alnus* sp., *Archontophoenixalexandrae*, *Arundinaria* sp., *Corylus* sp., *Cyatheadealbata*, *Podocarpusparlatorei*, *Polygonumchinense* and *Ripogonumscandens* (Samuels et al. 1987; Barr 1993; Hyde 1995; Candoussau et al. 1996; Hsieh et al. 1997; Taylor and Hyde 1999; Catania and Romero 2012; Li et al. 2015; Miller and Réblová 2021), but have never been reported as pathogens (Hyde et al. 2020a).

Samuels et al. (1987) accepted *Iodosphaeria* in Amphisphaeriaceae, and later, various authors placed it in Lasiosphaeriaceae and Trichosphaeriaceae (Barr 1990, 1994; Kang et al. 1998; Hilber and Hilber 2002; Jeewon et al. 2003). Again, Eriksson et al. (2001) placed *Iodosphaeria* in Amphisphaeriaceae. Later, Hilber and Hilber (2002) accommodated *Iodosphaeria* in the newly introduced family Iodosphaeriaceae. Maharachchikumbura et al. (2016) and Samarakoon et al. (2016) provided multigene phylogenies and accepted Iodosphaeriaceae in Xylariales. Hongsanan et al. (2017) treated it as Xylariomycetidae family *incertae sedis*, while Hyde et al. (2020a) and Wijayawardene et al. (2020) accepted Iodosphaeriaceae in Amphisphaeriales. In the most recent study of Miller and Réblová (2021), Iodosphaeriaceae is accounted as a family in Xylariales.

This study introduces three novel *Iodosphaeria* species from China, Taiwan, and Thailand. Detailed morphological descriptions, illustrations and a key are provided, and phylogenetic affinities of the new taxa are discussed.

## ﻿Materials and methods

### ﻿Morphological observations

Dead leaves were collected from Dahu Forest (Chiayi City, Taiwan) during autumn (September 2019), from dead twigs in Jinghong City (Yunnan Province, China) during winter (December 2019) and from dead leaves at MRC (Mushroom Research Centre, Chiang Mai, Thailand) during the rainy season (September 2020). Specimens were treated following the methods outlined in Senanayake et al. (2020). A Motic SMZ 168 Series microscope was used to examine fruiting structures. Hand sections of the fruiting structures were mounted in water and 5% KOH for microscopic studies and microphotography. Indian ink was used to stain any gelatinous sheath around the ascospores and Melzer’s reagent for ascal apical ring reaction. The micro-morphologies were examined using a Nikon ECLIPSE 80i compound microscope and photographed using a Canon 750D digital camera fitted to the microscope. Tarosoft (R) Image Frame Work program (IFW 0.97 version) and Adobe Photoshop CS6 software (Adobe Systems, USA) were used for image processing and measurements. The type specimens were deposited in the Mae Fah Luang University Herbarium (MFLU), Chiang Rai, Thailand and the Cryptogamic Herbarium, Kunming Institute of Botany Academia Sinica (HKAS), Chinese Academy of Sciences, Kunming, China. The new taxa were linked with Facesoffungi (Jayasiri et al. 2015) and Index Fungorum (http://www.indexfungorum.org).

### ﻿DNA extraction, PCR amplification and sequencing

DNA extraction, PCR amplification and sequencing were carried out following the methods described in Dissanayake et al. (2020). Direct DNA extraction was done using a Biospin Fungus Genomic DNA Extraction Kit-BSC14S1 (BioFlux, P.R. China) with 15–20 fruiting bodies of the fungus as described in Wanasinghe et al. (2018). PCR amplification was done using LSU and ITS DNA regions with LR0R/LR5 (Vilgalys and Hester 1990) and ITS5/ITS4 (White et al. 1990) primer pairs, respectively. The thermal cycling program was followed by Wanasinghe et al. (2020). Purified PCR products were sent to a commercial sequencing provider, Beijing Biomed Gene Technology Co., Ltd., Shijingshan District, TsingKe Biological Technology Co., Beijing, China.

### ﻿Phylogenetic analyses

**Figure 1.**
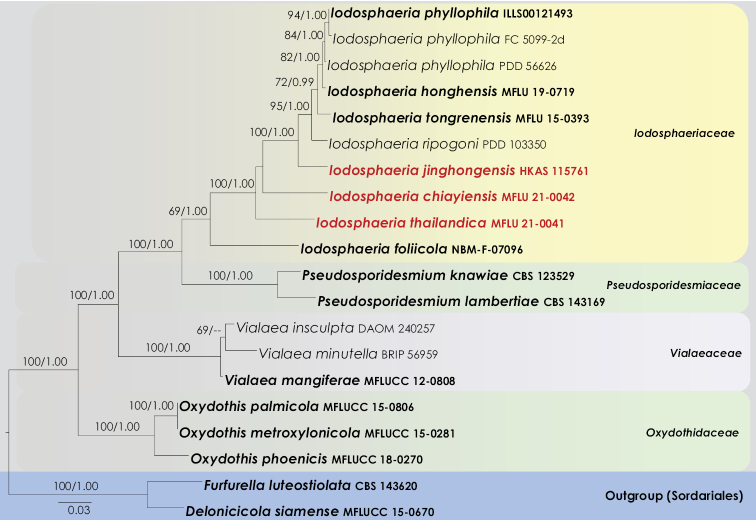
RAxML tree based on a combined dataset of partial LSU and ITS sequence analyses. The tree is rooted to *Delonicicolasiamense* (MFLUCC 15-0670) and *Furfurellaluteostiolata* (CBS 143620). Type strains are in bold, and the newly generated strains are in red.

Newly generated sequences were assembled and subjected to the standard BLAST search to identify the closest matches in GenBank. The accession numbers of taxa used in our analyses are shown in Table 1. Single datasets (LSU and ITS) were aligned using MAFFT v. 6.864b (http://mafft.cbrc.jp/alignment/server/index.html, Katoh and Standley 2013; Katoh et al. 2019), combined and manually improved using BioEdit v. 7.0.5.2 (Hall 1999). Maximum likelihood analysis and Bayesian inference (BI) were performed using RAxML-HPC2 on the XSEDE v. 8.2.10 tool and MrBayes 3.2.2 on the XSEDE tool in the CIPRES Science Gateway portal (Miller et al. 2012; Ronquist et al. 2012; Stamatakis 2014). The optimal ML tree was obtained with 1,000 separate runs under the GTR+GAMMA substitution model resulting from model tests using MrModeltest v. 2.3 (Nylander 2004) under the AIC (Akaike Information Criterion) implemented in PAUP v. 4.0b10. Maximum Likelihood bootstrap values (ML) equal or greater than 60% and Bayesian posterior probabilities (BYPP) equal or greater than 0.95 are presented above each node (Figure 1). All trees were visualized with FigTree v1.4.0 (Rambaut 2012), and the final layout was done with Microsoft PowerPoint (2016). The finalized alignment and tree were registered in TreeBASE (submission ID TB2: S29095). Reviewer access URL: http://purl.org/phylo/treebase/phylows/study/TB2:S29095?x-access-code=43fac9fe7622929c65c2bd4120a2c10a&format=html

**Table 1.** Taxa used in the phylogenetic analyses and corresponding GenBank accession numbers.

Taxon	Specimen/Strain	GenBank accession numbers		References
		ITS	LSU	
*Delonicicolasiamense*	MFLUCC 15-0670 T	MF167586	MF158345	Perera et al. (2017)
*Furfurellaluteostiolata*	CBS 143620 T	MK527842	MK527842	Voglmayr et al. (2019)
***Iodosphaeriachiayiensis***	**MFLU 21-0042** T	**MZ918994**	**MZ918992**	**This study**
*I.foliicola*	NBM-F-07096 T	MZ509148	MZ509160	Miller and Réblová (2021)
*I.honghensis*	MFLU 19-0719 T	MK737501	MK722172	Marasinghe et al. (2019)
***I.jinghongensis***	**HKAS 115761** T	**MZ918989**	**MZ923776**	**This study**
*I.phyllophila*	PDD 56626	MZ509149	MZ509149	Miller and Réblová (2021)
*I.phyllophila*	FC 5099-2d	MZ509150	N/A	Miller and Réblová (2021)
*I.phyllophila*	ILLS00121493 T	MZ509151	N/A	Miller and Réblová (2021)
*I.ripogoni*	PDD 103350	MZ509152	MZ509152	Miller and Réblová (2021)
***I.thailandica***	**MFLU 21-0041 T**	**MZ923759**	**MZ923758**	**This study**
*I.tongrenensis*	MFLU 15-0393 T	KR095282	KR095283	Li et al. (2015)
*Oxydothismetroxylonicola*	MFLUCC 15-0281 T	KY206776	KY206765	Konta et al. (2016)
*O.palmicola*	MFLUCC 15-0806 T	KY206774	KY206763	Konta et al. (2016)
*O.phoenicis*	MFLUCC 18-0270 T	MK088066	MK088062	Hyde et al. (2020a)
*Pseudosporidesmiumknawiae*	CBS 123529 T	FJ349609	FJ349610	Vu et al. (2019)
*P.lambertiae*	CBS 143169 T	MG386034	MG386087	Crous et al. (2017)
*Vialaeainsculpta*	DAOM 240257	KC181926	KC181924	McTaggart et al. (2013)
*V.mangiferae*	MFLUCC 12-0808 T	KF724974	KF724975	Senanayake et al. (2014)
*V.minutella*	BRIP 56959	JX139726	JX139726	Shoemaker et al. (2013)

Types strains are indicated with (T). Newly generated sequences are indicated in bold. “N/A” sequences are unavailable. Abbreviations: **BRIP**: Queensland Plant Pathology Herbarium, Australia; **CBS**: Centraalbueau voor Schimmelcultures, Utrecht, The Netherlands; **DAOM**: Plant Research Institute, Department of Agriculture (Mycology), Ottawa, Canada; **HKAS**: Chinese Academy of Sciences, Kunming, China. **KUMCC**: Kunming Institute of Botany Culture Collection, Chinese Academy of Science, Kunming, China; **MFLUCC**: Mae Fah Luang University Culture Collection, Chiang Rai, Thailand; **MFLU**: Mae Fah Luang University Herbarium, Chiang Rai, Thailand; **Others**: information not available.

## ﻿Results

### ﻿Phylogenetic analyses

The combined LSU and ITS comprise 20 taxa including the outgroup taxa. The best scoring RAxML tree is shown in Figure with a final ML optimization likelihood value of -7278.703992. The matrix had 575 distinct alignment patterns, with 19.44% undetermined characters or gaps. Estimated base frequencies were: A = 0.245534, C = 0.244177, G = 0.286855, T = 0.223434 substitution rates AC = 1.190714, AG = 2.269637, AT = 1.889784, CG = 1.069908, CT = 5.997198, GT = 1.000000; proportion of invariable sites I = 0.39717; gamma distribution shape parameters α = 0.578305. Both trees (ML and BYPP) were similar in topology and did not differ in species relationships, which is in agreement with multi-gene phylogenies of previous studies (Marasinghe et al. 2019; Miller and Réblová 2021).

In the combined multi-gene phylogenetic analysis, Iodosphaeriaceae received 100% ML and 1.00 BYPP support values (Figure 1). Three strains of *Iodosphaeriaphyllophila* grouped as a monophyletic clade with 82% ML and 1.00 BYPP support. *Iodosphaeriahonghensis* (MFLU 19-0719) nested as a sister clade to *I.phyllophila* with 82% ML and 1.00 BYPP support. Within the *Iodosphaeria* clade, our new collections *viz.*HKAS 115761 (*I.jinghongensis*), MFLU 21-0042 (*I.chiayiensis*) and MFLU 21-0041 (*I.thailandica*) grouped as distinct lineages (Figure 1). *Iodosphaeriajinghongensis* was distinct from *I.ripogoni* by 100% ML and 1.00 BYPP support values. *Iodosphaeriachiayiensis* nested between *I.thailandica* and *I.jinghongensis*. However, this relationship is statistically not supported. *Iodosphaeriathailandica* received 100% ML and 1.00 BYPP support values. *Iodosphaeriafoliicola* (NBM-F-07096) is grouped as the basal taxon in the Iodosphaeriaceae.

## ﻿Taxonomy

### ﻿ Iodosphaeria chiayiensis

Taxon classificationFungiXylarialesIodosphaeriaceae

Marasinghe, C.H. Kuo & K.D. Hyde, sp. nov.

B66F83E0-54D9-5E0F-91BD-1AA0A819845C

IndexFungorum number: IF558412

Facesoffungi Number No: FoF09711

Figure 2

#### Etymology.

The specific epithet *chiayiensis* refers to the city name where the fungus was collected.

#### Holotype.

MFLU 21-0042.

#### Description.

*Saprobic* on dead leaves of an unidentified host. **Sexual morph**: *Ascomata* 150–190 × 160–200 μm (x̅ = 170 × 180 μm, n = 10), globose to subglobose, superficial, black, solitary to gregarious, consisting of numerous long, flexuous setae. *Setae* 3–5 μm wide, arising from cells at the peridium surface, brown, unbranched, septate, apex flattened. *Ostiole* periphysate, apapillate. *Peridium* 50–55 μm wide (x̅ = 53.4 μm, n = 10), comprises two layers of *textura angularis* cells, outer layer of dark brown to black thick-walled cells, and an inner layer of flattened, light brown. *Paraphyses* 2–4 μm wide, shorter than asci, hyaline, embedded in a gelatinous matrix. *Asci* 60–90 × 8–10 μm (x̅ = 72.9 × 9.2 μm, n = 30), 8-spored, unitunicate, cylindrical, shortly pedicellate, apex rounded, with a J+ apical ring. *Ascospores* 15–20 × 4–6 μm (x̅ = 17.2 × 5.2 μm, n = 30), overlapping uni-seriate, ellipsoidal to allantoid, aseptate, hyaline, guttulate. **Asexual morph**: Undetermined

**Figure 2.**
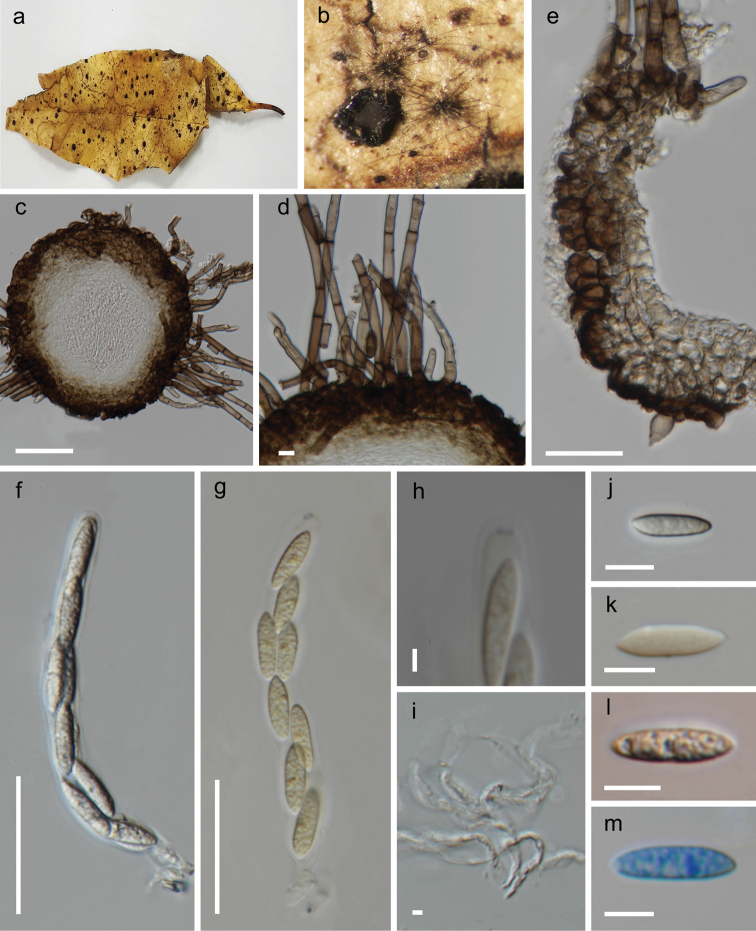
*Iodosphaeriachiayiensis* (MFLU 21-0042, holotype) **a** substrate **b** ascomata on the host surface **c** section of ascoma **d** appearance of setae on peridium **e** peridium **f, g** asci **h** J+ apical ring (in Melzer’s reagent) **i** paraphyses **j–m** ascospores (**m** stained in lactophenol cotton blue). Scale bars: 50 μm (**c, e**); 5 μm (**d**); 20 μm (**f–g**); 5 μm (**h, i**); 10 μm (**j–m**).

#### Material examined.

Taiwan, Chiayi, Fanlu Township area, on dead leaves of an undetermined species, 10 September 2019, D.S Marasinghe, DTF018 (MFLU 21-0042, ***holotype***).

#### Notes.

*Iodosphaeriachiayiensis* resembles *I.polygoni* which has globose to sub globose, superficial, solitary to gregarious ascomata, cylindrical, short pedicellate asci with J+, apical rings and ellipsoidal to allantoid, aseptate, guttulate ascospores. However, *I.chiayiensis* differs from *I.polygoni* in having smaller ascomata (150–190 × 160–200 μm *vs.* 270–475 × 250–500 μm) and shorter asci (60–90 × 8–10 μm *vs.* 150–180 × 10–13 μm) (Hsieh et al. 1997). In the multi-gene phylogenetic analyses (Figure 1), our collection (*Iodosphaeriachiayiensis*, MFLU 21-0042) has close affinity to *I.thailandica.* However, it was not possible to compare *I.chiayiensis* and *I.jinghongensis* as they occur as different morphs.

### ﻿ Iodosphaeria jinghongensis

Taxon classificationFungiXylarialesIodosphaeriaceae

L.S. Dissan., J.C. Kang & K.D. Hyde, sp. nov.

7980BE84-B486-5FBC-8D5A-09371013AA6D

IndexFungorum number: IF558800

Facesoffungi Number No: FoF09712

Figure 3

#### Etymology.

The specific epithet *jinghongensis* refers to the city name where the fungus was collected.

#### Holotype.

HKAS 115761.

#### Description.

*Saprobic* on dead twigs of an unidentified host. **Sexual morph**: Undetermined. **Asexual morph**: Colonies on natural substrate effuse, punctiform, scattered, blackish brown, mycelium mostly superficial, non-branched, hyaline, smooth hyphae. *Conidiophores* micronematous, smooth, flexuous, pale brown. *Conidia* ceratosporium-like, arising from aerial hyphae, solitary, dry, composed of a central cell and 2–3 arms. *Arms* 70–93 × 9–14 μm (x̅ = 79.8 × 12.1 μm, n = 20), wide at the tip 5–8 μm (x̅ = 6.9 μm), radiating from the centrally located attachment point, multi-septate (9–10), each septum with a central pore, brown, often with a sub-globose to conical cell at the point of attachment, dehiscence scar circular 3–4 μm diam. (x̅ = 3.5 μm).

**Figure 3.**
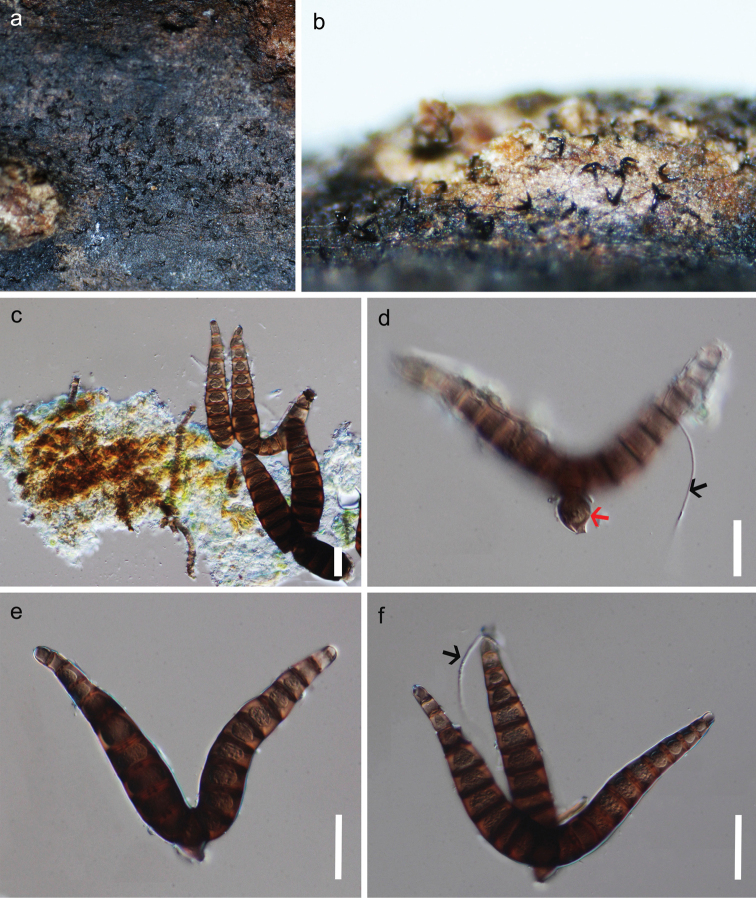
*Iodosphaeriajinghongensis* (HKAS 115761, holotype) **a, b** colonies on the host surface **c–f** conidia, conidiogenous cells and conidiophores (black arrow shows hyphae, red arrow shows conidiophore). Scale bars: 20 μm (**c–f**).

#### Material examined.

China, Yunnan Province, Xishuangbanna Dai Autonomous Prefecture, Jinghong City, Jinghaxiang (21°780617'N, 101°056122'E), on a dead twig of undetermined species, 19 December 2019, D.N. Wanasinghe, DW060 (HKAS 115761, ***holotype***).

#### Notes.

**Figure 4.**
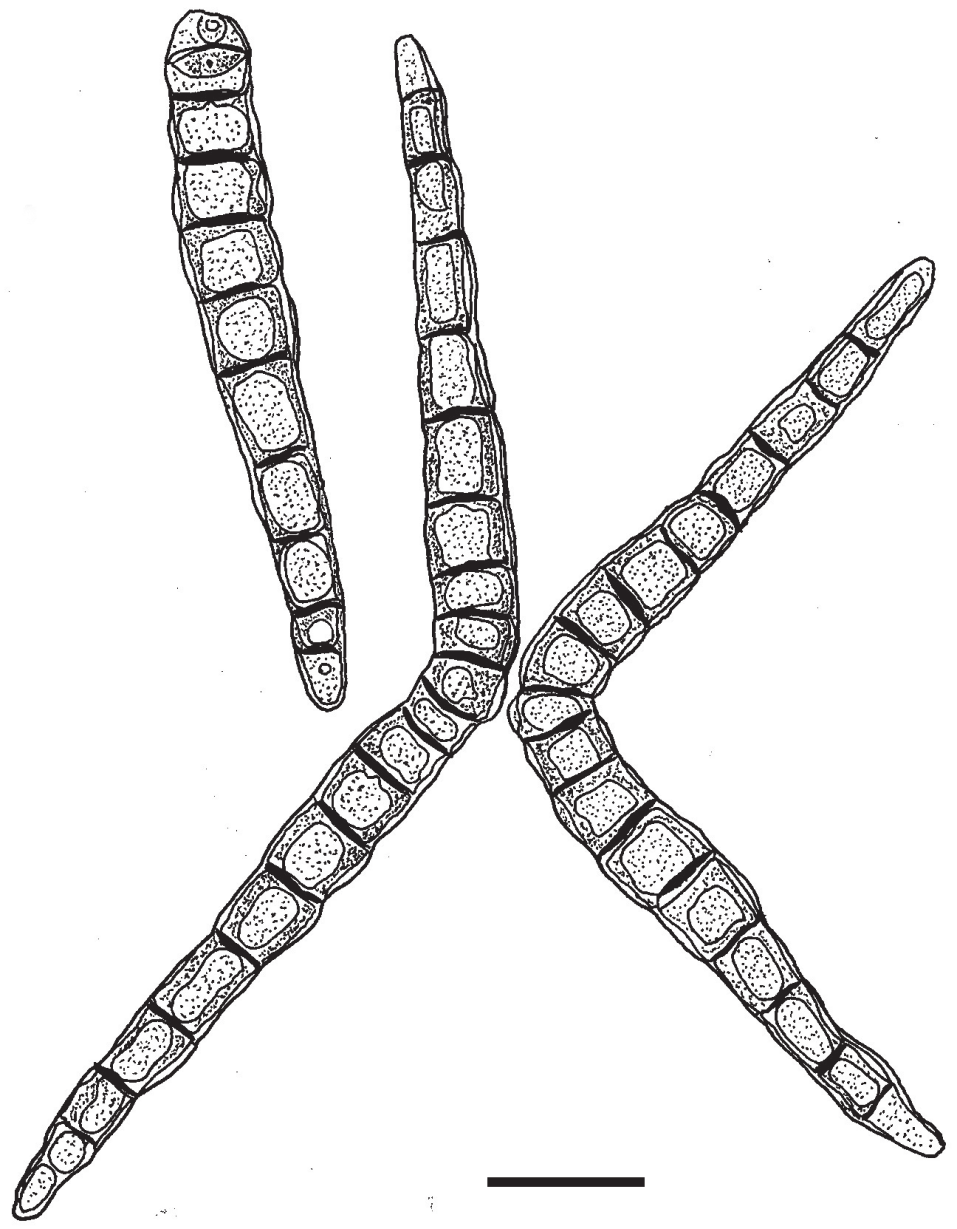
Asexual morph of *Iodosphaeriaripogoni* (ceratosporium-like conidia). Redrawn from: Samuels et al. (1987). Scale bar: 20 μm.

*Iodosphaeriajinghongensis* is similar to *I.ripogoni* in having septate, brown, subglobose to conical conidia with 2–3 arms (Figure 4; Samuels et al. 1987). However, *I.jinghongensis* differs from *I.ripogoni* in having smaller arms (70–93 × 9–14 μm *vs* 95–120 × 14–16 μm). *Iodosphaeriaripogoni* was collected from the stem of *Ripogonumscandens* from New Zealand, and *I.jinghongensis* was collected from twigs of undetermined species from China.

### ﻿ Iodosphaeria thailandica

Taxon classificationFungiXylarialesIodosphaeriaceae

L.S. Dissan., Marasinghe, & K.D. Hyde, sp. nov.

BCD7BA98-E309-5688-9ECD-67D1321BA46A

IndexFungorum number: IF558411

Facesoffungi number No: FoF09710

Figure 5

#### Etymology.

The specific epithet *thailandica* refers to the country where the fungus was collected.

#### Holotype.

MFLU 21-0041

#### Description.

*Saprobic* on dead leaves of unidentified host. **Sexual morph**: *Ascomata* 250–285 × 250–295 μm (x̅ = 267.3 × 272 μm, n = 10), globose to subglobose, superficial, black, solitary to gregarious, consisting of numerous long, flexuous setae. *Setae* 4.5 μm wide, arising from cells at the peridium surface, dark brown to brown, unbranched, septate. *Ostiole* periphysate, apapillate. *Peridium* 40–50 μm wide (x̅ = 44.6 μm, n = 10), comprising two layers of cells of *textura angularis*, outer layer of dark brown to black thick-walled cells and an inner layer of flattened, hyaline cells. *Paraphyses* 5–8 μm wide, length as longer than asci, septate, hyaline, branched, embedded in a gelatinous matrix. *Asci* 65–100 × 8–10 μm (x̅ = 84.3 × 8.9 μm, n = 30), 8-spored, unitunicate, cylindrical, short pedicellate, apex rounded, with a J+ apical ring. *Ascospores* 20–35 × 2–4 μm (x̅ = 29.1 × 3.2 μm, n = 30), overlapping uni-seriate, cylindrical to allantoid, aseptate, hyaline to pale brown, guttulate, slightly curved. **Asexual morph**: Undetermined.

**Figure 5.**
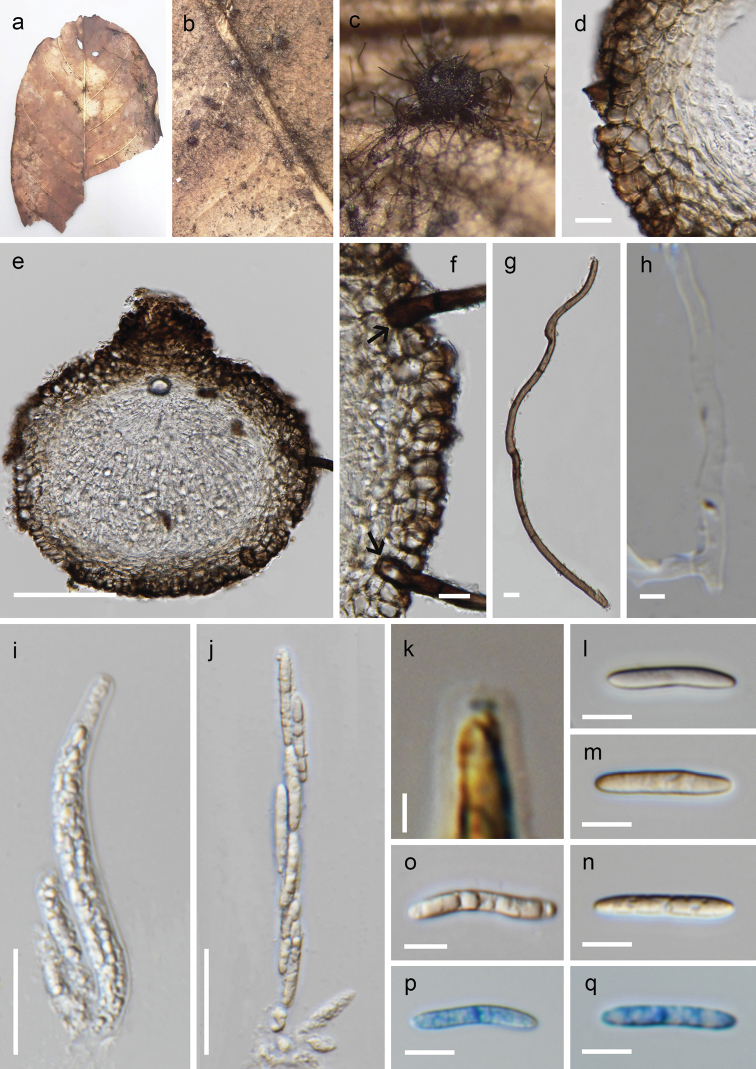
*Iodosphaeriathailandica* (MFLU 21-0041, holotype) **a** substrate **b, c** ascomata on the host surface **d** peridium **e** section of ascomata **f** appearance of setae (black arrow) on peridium **g** setae **h** paraphyses **i, j** asci **k** J+ apical ring (in Melzer’s reagent) **l–q** ascospores (**p, q** stained in Lactophenol Cotton Blue). Scale bars: 10 μm (**d**); 100 μm (**e**); 5 μm (**f–h**); 20 μm (**i, j**); 10 μm (**k–q**).

#### Material examined.

Thailand, Chiang Mai, Mushroom Research Centre, on dead leaves of an undetermined species, 11 September 2020, D.S Marasinghe, DMRC011 (MFLU 21-0041, ***holotype***)

#### Notes.

*Iodosphaeriathailandica* shares similar characteristics with *I.honghensis* in having globose to subglobose, superficial, solitary to gregarious ascomata, cylindrical, short pedicellate, J+, apical ring and cylindrical to allantoid asci with aseptate, guttulate ascospores (Marasinghe et al. 2019). However, *I.thailandica* differs from *I.honghensis* in having long, narrow (20–35 × 2–4 μm) and hyaline to pale brown ascospores versus short, broad (18.5–22.5 × 4.5–6.5 μm) and hyaline ascospores. In the phylogenetic analyses, *I.thailandica* is distinct from other species in the genus by 100 % ML and 1.00BYPP and sister to the *I.chiayiensis*. *Iodosphaeriathailandica* has larger ascomata (250–285 × 250–295 μm), cylindrical to allantoid asci and hyaline to pale brown ascospores, while the ascomata of *I.chiayiensis* are smaller (150–190 × 160–200 μm) and ascospores are hyaline and ellipsoidal to allantoid. *Iodosphaeriathailandica* is the first report of *Iodosphaeria* from Thailand.

### ﻿Key to the accepted *Iodosphaeria* species based on known sexual morph

**Table:** 

1	Asci with a distinct apical ring	**2**
–	Asci lacking a distinct apical ring	**10**
2	Apical ring not staining blue in Melzer’s reagent	***I.arundinariae***
–	Apical ring staining blue in Melzer’s reagent	**3**
3	Ascomata immersed to erumpent	***I.aquatica***
–	Ascomata superficial	**4**
4	Ascospores guttulate	**5**
–	Ascospores eguttulate	**8**
5	Ascospores ellipsoidal	**6**
–	Ascospores cylindrical	**7**
6	Ascomata 270–475 × 250–500 μm	***I.polygoni***
–	Ascomata 150–190 × 160–200 μm	***I.chiayiensis***
7	Ascospores 18.5–22.5 × 4.5–6.5 μm, hyaline	***I.honghensis***
–	Ascospores 20–35 × 2–4 μm, hyaline to pale brown	***I.thailandica***
8	Asci shorter than 150 μm	**9**
–	Asci longer than 150 μm	***I.tongrenensis***
9	Ascospores allantoid	**11**
–	Ascospores ellipsoidal	***I.podocarpi***
10	Ascospores with a mucilaginous sheath	***I.ripogoni***
–	Ascospores without a mucilaginous sheath	***I.hongkongensis***
11	Paraphyses of similar length to asci	***I.foliicola***
–	Paraphyses longer than asci	***I.phyllophila***

## ﻿Discussion

*Iodosphaeria* is seldom collected. In 15 years of studying fungi in Hong Kong, only a single collection was found despite intensive collection efforts (Taylor and Hyde 1999). *Iodosphaeria* is widely distributed in temperate and tropical regions, e.g., China (Guizhou, Yunnan), Europe (Belgium, Germany), Great Britain, Canada, Hong Kong, New Zealand, South America (Brazil, Argentina, French Guiana), Taiwan and USA (Louisiana) (Samuels et al. 1987; Barr 1993; Hyde 1995; Candoussau et al. 1996; Hsieh et al. 1997; Taylor and Hyde 1999; Catania and Romero 2012; Li et al. 2015; Marasinghe et al. 2019; Miller and Réblová 2021). This genus is saprobic on dead plant substrates in terrestrial grassland habitats (Barr 1993), on fern rachides (Samuels et al. 1987), on dead petioles of palms (Taylor and Hyde 1999), and on submerged wood in freshwater (Hyde 1995) but has never been reported as pathogenic on hosts. They are likely endophytes that become saprobes during leaf senescence (Hyde et al. 2020a). *Iodosphaeria* species may not be host-specific due to their wide distribution range (Miller and Réblová 2021). The genus may be much more diverse than presently known, as is true for many other microfungal genera (Hyde et al. 2020b).

The asexual morphs of this genus were recorded as selenosporella- or ceratosporium-like (Samuels et al. 1987; Li et al. 2015; Marasinghe et al. 2019). *Iodosphaeriaphyllophila*, *I.polygoni* and *I.ripogoni* (Figure 4) were introduced with both sexual and asexual morphs (Hsieh et al. 1997; Samuels et al. 1987). *Iodosphaeriahonghensis* and *I.tongrenensis* were observed to have ceratosporium-like conidia on their host surface (Li et al. 2015; Marasinghe et al. 2019). Samuels et al. (1987) observed another asexual morph of selenosporella- like conidia that was different from ceratosporium-like conidia. In present study, we establish ceratosporium-like conidia as an asexual morph of *Iodosphaeria*.

## Supplementary Material

XML Treatment for Iodosphaeria chiayiensis

XML Treatment for Iodosphaeria jinghongensis

XML Treatment for Iodosphaeria thailandica
